# Adolescents living with HIV are at higher risk of death and loss to follow up from care: Analysis of cohort data from eight health facilities in Ethiopia

**DOI:** 10.1371/journal.pone.0223655

**Published:** 2019-10-17

**Authors:** Degu Jerene, Workeabeba Abebe, Kefyalew Taye, Andrea Ruff, Inger Hallstrom

**Affiliations:** 1 KNCV Tuberculosis Foundation, The Hague, The Netherlands; 2 Addis Ababa University, Department of Pediatrics and Child Health, Addis Ababa, Ethiopia; 3 Hawassa University, Department of Pediatrics and Child Health, Hawassa, Ethiopia; 4 Johns Hopkins University, Bloomberg School of Public Health, Department of International Health, Baltimore, Maryland, United States of America; 5 Lund University, Faculty of Medicine, Department of Health Sciences, Lund, Sweden; Yeshiva University Albert Einstein College of Medicine, UNITED STATES

## Abstract

**Background:**

There are limited data on the treatment outcomes of adolescents living with HIV. Our objective was to compare mortality and loss to follow up (LTFU) rates between adolescent and younger age groups at enrollment in care.

**Methods:**

This was a retrospective cohort study carried out in eight health facilities in two regions of Ethiopia. Adolescents (age 10–14 and 15–19 year) and children (age 0–9 year) enrolled in chronic HIV care between 2005 and 2013 constituted the study population. We reviewed the individual patient charts between March and June 2014 and updated the data on the status of each patient through December 2015. We used death and loss-to-follow up as primary endpoints and used the Cox-regression analysis where age, categorized as adolescent versus child, was the main predictor variable.

**Results:**

Of 2058 participants studied, 52.1% were adolescents. The cohort contributed 2422 person-years of observation (PYO) during the pre-ART follow-up, whereas 1531 patients put on ART contributed 5984 PYO. Of those put on ART, 209 (13.7%) LTFU and 92 (6%) deaths were reported. Adolescents in age group 15–19 yr had the highest risk of LTFU [adjusted hazard ratio, aHR (95% CI) = 3.1 2.1, 5.0 ] followed by those in age group 10–14 yr (aHR = 1.5 [0.9, 2.3]) compared with children aged 0–9 yr. Mortality hazard was significantly higher among younger adolescents (aHR = 2.8 [1.4, 5.4]) and older adolescents (aHR = 2.3 [1.1, 4.9]) compared with children.

**Conclusions:**

Adolescents are at higher risk of mortality and LTFU as compared to children ages 0–9. Younger adolescents and children had similar LTFU rates. Narrow age band disaggregated analysis can serve as useful guide for tailoring interventions to the specific needs of different age groups.

## Introduction

Important health problems and risk factors for adult disease emerge in young people aged 10–24 years which comprise over a quarter of the world’s population [1]. However, the health problems of this age group are often neglected because of the perception that this age group represents healthy population. Adolescence, being a unique period in an individual’s course of life, requires a special attention in health programming. Adolescents are at heightened risk of a number of health problems including HIV, substance use, injuries, mental health, sexual and reproductive health, and violence, warranting the need for multipronged interventions [2].

When the Joint United Nations Programme on HIV/AIDS (UNAIDS) and partners set the 90-90-90 ambitious fast-track target of HIV testing, treatment initiation and viral suppression, children and adolescents were considered [3]. However, the 2018 UNAIDS progress update indicates that in Sub-Saharan Africa, young women and adolescent girls bear the brunt of weaknesses in HIV prevention efforts in the continent [4]. Despite constituting only 10% of the population, 25% of all new infections in this sub-region was among young women and young girls. Also, HIV was among the top 10 leading causes of mortality among adolescents [4]. Structural barriers such as restrictive laws on the age of consent as well as health systems factors such as poor quality of services contribute to high rates of poor treatment outcomes [5]. The problem of poor treatment adherence among adolescents has been noted as global priority in several recent reports [6] which highlighted the need for generating more evidence to guide contextualized interventions.

Many adolescents acquire HIV sexually but with the improved availability of antiretroviral therapy (ART) [7], increasing number of vertically infected children are growing to adolescence and adult hood [8]. Such large number of HIV infected adolescents were not expected during the early phase of the global HIV epidemic because of the expectation that vertically infected children would not survive to adolescence and adulthood [9]. More recent global cohort data on adolescents aged 10–14 years showed that about 79% of perinatally HIV infected adolescents were living in sub-Saharan Africa [10]. This collaborative cohort analysis also demonstrated that both mortality and lost to follow up (LTFU) rates are higher among perinatally HIV infected adolescents from sub-Saharan Africa compared with those from other continents.

Some of the earlier studies from resource-limited settings compared treatment outcomes between adolescents and other age groups but outcomes varied considerably from study to study. In a South African cohort, for example, virologic failure rate was higher among adolescents than young adults, immunological response was greater in adolescents, whereas mortality and LTFU rates were similar in both groups [11]. On the other hand, Nachega et al from the same country reported lower rates of immunologic recovery and higher rates of virologic rebound after initial suppression but confirmed poorer adherence rates among adolescents [12]. A study among Haitian adolescents and youth demonstrated that they were at high risk of virologic failure and disease progression [13]. A Ugandan study showed similar mortality rates among children, adolescents and adults [14]. A more recent collaborative cohort analysis suggested that mortality rates were higher among perinatally infected adolescents receiving ART in lower income and lower-middle income countries compared with those from high income countries. On the other hand, LTFU rates were higher among adolescents from high income countries [15].

Studies showing the full spectrum of attrition rates among African patient populations are still limited. This is entirely lacking in Ethiopia. In this study, we evaluated rates of mortality and LTFU among adolescents as compared to children ages 0–9, and we analyzed factors associated with LTFU and mortality among adolescents living with HIV.

## Methods

### Study design and setting

This was a retrospective cohort study based on review of the clinical records of patients treated in eight public health facilities in two regions of Ethiopia. The two regions, Addis Ababa and Southern Nations', Nationalities' and Peoples' Region (SNNPR), were selected for two reasons. First, the authors had previous experience working with the regions where there were easily accessible, high patient load centers. Secondly, it enabled the study team to compare treatment outcomes from the perspective of two contrasting regions. Addis Ababa is a major urban setting with an estimated HIV prevalence of 5.2% whereas SNNPR is a predominantly rural region with a very low HIV prevalence (0.9%) at the time of data collection [16].

All sites had standardized service packages according to the national guidelines. They had stand-alone HIV services within the broader health service facility. All sites had adolescent-friendly services, introduced through external support. The difference between SNNPR and Addis Ababa sites is that Addis Ababa, being a capital city, has the added advantage of being cared for by specialist physicians when needed.

### Study participants

Patients enrolled into the chronic HIV care between 2005 and 2013 in the eight selected health facilities (seven hospitals and one health center) served as source population. Adolescents and children who fulfilled the following criteria were included in the study:

- Adolescents (10–19 yrs) enrolled with the HIV clinics of the below sites between the period Oct 10, 2005-September 9, 2013.
- Comparative group of children 0–9 years enrolled over the same period.
- Those who had had at least two documented clinic visits.
- ART-naïve at enrollment in the study clinic.

Those who were transferred-in from other health facilities and patients with incomplete documentation of basic socio-demographic characteristics (age, sex, and place of residence) were excluded. Age at enrolment in pre-ART care was considered in the assignment of age group. Growing into adolescence during follow-up was not considered.

### Sampling and sample size

We used the list of patients in the ART clinic registers as a sampling frame. At each health facility, an age- stratified list of patients was generated to serve as a sampling frame. For the purpose of sampling, we stratified the sampling frame into two groups: adolescent (10–19 yr) and child (0–9 yr). We then applied a systematic random sampling approach until a predetermined sample size was achieved. Sample size per site was determined proportionate to the patient population size. We took a combined pre-ART and on-ART LTFU rate as the main outcome variable. Hypothesizing that adolescents are exposed groups with higher LTFU rates, we considered the following assumptions to arrive at a minimum sample size of 718 per group: CI of 95%; Power 90%; Ratio of exposed to unexposed: 1; 10% of the unexposed will be lost to follow up; Odds ratio of 1.8; and 20% non-response rate. We used OpenEpi software (available at http://openepi.com/Menu/OE_Menu.htm) for computing the sample size.

### Data collection

Data collection took place between March-June 2014. Since this was a retrospective study, we used patient charts, pre-ART registers, and ART registers as main data sources. A nurse, assisted by two data clerks collected the data using a pre-tested data abstraction form. All completed data abstraction forms were submitted to co-investigators who were based at each region who then checked for errors and omissions before submitting to the primary author. The primary author did further check of submitted paper data forms for completeness and then stored at secure place. The primary author checked each stored data form before it was entered by a central data entry clerk. Further, the primary author checked each entered data on a regular basis.

### Key predictor and outcome variables and definitions

Patients’ age group categorized as adolescent versus child was the main predictor variable of interest. We further stratified the adolescent age group into 10–14 yr and 15–19 yr based on the emerging evidence that showed significant differences in treatment outcomes between the two age groups. Baseline WHO clinical stage, CD4 count, Hemoglobin (Hgb), sex, region, and place of residence were the co-variables used. We used death and LTFU after ART initiation as primary outcome variables. Since viral load was rarely done during the study period, we did not include it either as co-variate or outcome variable.

We defined pre-ART and post-ART outcomes a priori, as done in our previous adult cohort studies [17]. Accordingly, we defined (pre-) ART outcomes as: (a) ‘still under pre-ART care’-if patient was registered with the ART clinic of the hospital, had regular follow-up with the clinic and was not having follow-up at another health facility; (b) ‘lost to follow-up, ‘-if patient did not have follow-up visit at least 30 days after the last date of the next clinic appointment; (c) ‘put on ART’-if patient was started on ART in the hospital clinic; (d), ‘died before starting ART’-if patient was known to be dead as recorded by treating clinicians; and (e), ‘transferred out’-if patient moved to another health facility with confirmed written documentation of transfer out. We did not perform extended community-based tracing of LTFU patients due to resource limitation. We also did not attempt to ascertain specific causes of death for the same reason.

### Data analysis

We described baseline characteristics of the cohort and calculated death and LTFU rates first for the entire cohort and then for the three age groups. Variables found to be associated with increased risk of LTFU or death in univariate analysis (P<0.25) were included in a cox regression survival analyses for adjusting for potential confounders. We used hazard rations (HR) with 95% CI to assess statistical significance which was determined to be P<0.05. Survival curves were used to further illustrate differences in HR. In addition, we used Kaplan-Meier analyses to highlight important features of potential confounders. The log-rank test was used to determine the statistical significance of the Kaplan-Meier curves. Because of the retrospective nature of the study, missing data was anticipated. We checked for patterns of missing data and list-wise deletion method was used to handle missing variables. Statistical Package for Social Sciences (SPSS) version 22.0 was used for data entry and analysis.

### Ethical considerations

Since this was retrospective record review, informed consent from individual patients was not feasible. The records, however, were accessed with written permission from the heads of respective health facilities. Also, patient identifiers were not included in the final data file that was analysed to ensure anonymity. Moreover, data security was ensured by keeping completed data abstracts in a lockable shelf only accessible for the primary author. Although the study has no direct benefit to the study participants, its potential benefit to the wider population of adolescents living with HIV was communicated to health facility staff and heads. The study protocol was reviewed and approved by the Institutional Review Board of College of Health Sciences, Addis Ababa University and by Ethics Committees of respective regional health bureaus, and by the National Research Ethics Committee of Ethiopia. All research staff were trained on the ethical conduct of Human Subjects Research.

## Results

### Description of participants

Of 2058 eligible participants included in the study, females constituted 53.4% of the participants and most (87.7%) were from urban areas.

There were statistically significant differences among the three age groups in all baseline characteristics assessed except for WHO clinical stage. With regard to regional representation, majority of older adolescents (69.1%) were from SNNPR while the reverse was true for younger adolescents (69.4% from Addis Ababa). Table 1 describes baseline characteristics of the participants by three age groups. Of 986 children aged 0–9 years, 246 (25%) were aged two years and younger, (486 (49.2%) were between 0–4 years, and 500 (50.7%) were between 5–9 years of age (data not shown in table).

**Table 1:** Baseline characteristics of the participants.

Characteristic	Age group, N(%)				Chi-sq; P-value
	0–9 yr	10–14 yr	15–19 yr	Total	
Sex					38.1; P<0.001
Male (%)	526 (53.3)	335 (51.5)	92 (22)	953 (46.3)	
Female (%)	456 (46.2)	315 (48.4)	329 (78)	1100 (53.4)	
Missing	4 (0.4)	1 (0.2)	0	5 (0.2)	
Total	986 (100)	651 (100)	421(100)	2058 (100)	
Residence					
Urban	870 (88)	567 (87.1)	367 (87.2)	1804 (87.7)	4.9; P<0.05
Rural	113 (11)	44 (6.8)	43 (10.2)	199 (9.7)	
Missing	3 (0.3)	40 (6.1)	11 (2.6)	55 (2.7)	
Total	986 (100)	651 (100)	421 (100)	2058 (100)	
Region					
Addis Ababa	446 (45.2)	452 (69.4)	130 (30.9)	1028 (50)	16.9; P<0.001
SNNPR	540 (54.8)	199 (30.6)	291 (69.1)	1030 (50)	
Total	986 (100)	651 (100)	421 (100)	2058 (100)	
WHO clinical stage					
I-II	491(49.8)	309 (47.5)	192 (45.6)	992 (48.2)	13; P>0.05
III-IV	454 (46.0)	328 (50.4)	216 (51.3)	998 (48.5)	
Missing	41 (4.2)	14 (2.2)	13 (3.1)	68 (3.3)	
Total	986 (100)	651(100)	421 (100)	2058 (100)	
CD4 count					
<350	305 (30.9)	433 (66.5)	260 (61.7)	998 (48.5)	223; P<0.001
> = 350	599 (60.9)	194 (29.8)	135 (32.1)	928 (45.1)	
Missing	82 (8.3)	24 (3.7)	26 (6.2)	132 (6.4)	
Total	986 (100)	651(100)	421 (100)	2058 (100)	
Hgb					
<10g/dl	115 (11.7)	48 (7.4)	35 (8.3)	198 (9.6)	11.9; P<0.01
> = 10g/ml	459 (46.6)	376 (57.8)	205 (48.7)	1040 (50.5)	
Missing	412 (41.8)	227 (34.8)	181 (43.0)	820 (39.8)	
Total	986 (100)	651 (100)	421(100)	2058 (100)	

The median age of children 0–9 yr (IQR) was 5 (2–7); median age of older adolescents (IQR) was 13 (11–18), and those of younger adolescents was 12 (10–13).

On further bivariate analysis of adolescents and children who were started on ART, there were more rural residents among older adolescents (7.9%) and children (9%) compared with younger adolescents (4.5%) and the difference was statistically significant (p<0.001). Most of the older adolescents (63.2%) and majority of children (54.7%) were from SNNPR while majority of younger adolescents (75.9%) were from Addis Ababa. Also, there were more females in the older adolescent age group (76.5%) compared with younger adolescents (49.2%) and children (54.3%). On the other hand, there was no difference among the three groups in terms of WHO clinical stage. Table 2 describes baseline characteristics of those started on ART by age group.

**Table 2:** Baseline characteristics of patients who were put on ART.

Characteristic		Age group, N(%)				Chi-sq; P-value
		0–9 yr	10–14 yr	15–19 yr	Total	
Sex	Male	388 (54.3)	273 (50.6)	65 (23.5)	726 (47.4)	38.6; p<0.001
	Female	323 (45.2)	265 (49.2)	212 (76.5)	800 (52.3)	
	Missing	4 (0.6)	1 (0.2)	0	5 (0.3)	
	Total	715 (100)	539 (100)	277 (100)	1531 (100)	
Residence	Urban	648 (90.6)	476 (88.3)	245 (88.4)	1369 (89.4)	80.7; p<0.001
	Rural	64 (9)	24 (4.5)	22 (7.9)	110 (7.2)	
	Missing	3 (0.3)	39 (7.2)	10 (3.6)	52 (3.4)	
		715 (100)	539 (100)	277 (100)	1531 (100)	
Region	Addis Ababa	391 (54.7)	409 (75.9)	102 (36.8)	902 (58.9)	125; p<0.001
	SNNPR	324 (45.3)	130 (24.1)	175 (63.2)	629 (41.1)	
	Total	715 (100)	539 (100)	277 (100)	1531 (100)	
WHO stage	I-II	237 (33.1)	189 (35.1)	86 (31)	512 (33.4)	1.3; P>0.1
	III-IV	467 (65.3)	347 (64.4)	189 (68.2)	1003 (65.5)	
	Missing	11 (1.5)	3 (0.6)	2 (0.7)	16 (1)	
	Total	715 (100)	539 (100)	277 (100)	1531 (100)	
CD4 value	<350	393 (55)	478 (66.9)	250 (90.3)	1121 (73.2)	187; p<0.001
	> = 350	269 (37.6)	59 (8.3)	23 (8.3)	351 (22.9)	
	Missing	53 (7.4)	2 (0.3)	4 (1.4)	59 (3.9)	
	Total	715 (100)	277 (100)	277 (100)	1531 (100)	
Hgb	<10g/dl	115 (16.1)	48 (8.9)	35 (12.6)	198 (12.9)	14.2; p<0.01
	> = 10g/dl	459 (64.2)	376 (69.8)	205 (74)	1040 (67.9)	
	Missing	141 (19.7)	115 (21.3)	37 (13.4)	293 (19.1)	
	Total	715 (100)	539 (100)	277 (100)	1531 (100)	

### Pre-ART period description

The cohort contributed 2422.44 person-years of observation (PYO) during the pre-ART follow-up, with the median (interquartile range, IQR) follow-up time being 0.24 (0.02–1.58) person-years. The pre-ART duration was shorter in adolescents than in children (61.5% of adolescents versus 38.5% of children had pre-ART duration below the median; p<0.001). At the end of the pre-ART follow-up period, 310 (15.1%) patients were lost to follow-up while only 12 (0.6%) patients were reported to have died. There was no statistically significant difference between adolescents and children in terms of pre-ART outcomes (P = 0.071).

### Post-ART period description

**Fig 1:**
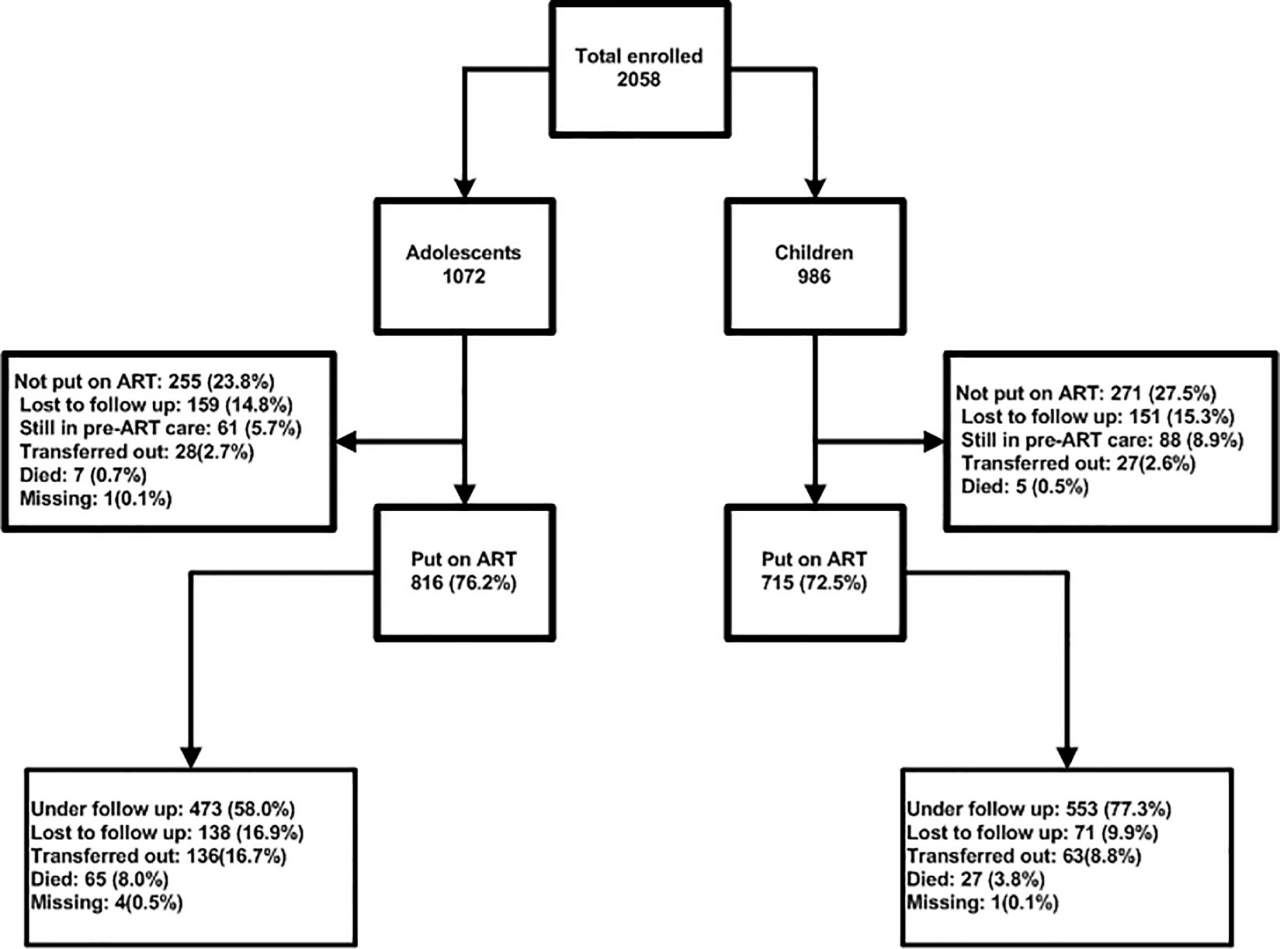
Cohort profile and follow-up status of patients.

During after-ART follow up, 1531 patients put on ART contributed 5984.04 PYO, and the median (IQR) follow-up period was 3.79 (1.42–6.15) years. At the end of the follow-up period, 209 (13.7%) LTFU and 92 (6%) deaths were reported, giving a combined attrition rate of 19.7%. Both LTFU (17% versus 9.9%) and death (8% versus 3.8%) were higher in adolescents than in children (p<0.001). When further stratified by the three age groups, only 51.6% of older adolescents were under follow-up compared with 77.5% among children and 61.7% among younger adolescents. The proportion LTFU was 24.5%, 13.1% and 9.9% in older adolescents, younger adolescents and children respectively. The proportion died was 8.8% in younger adolescents, 7.6% in older adolescents and 3.8% in children. Fig 1 summarizes cohort profile and follow up status of patients.

### Rates and predictors of loss to follow-up and mortality after ART initiation

The overall rate of LTFU was 3.49 per 100 PYO [95% CI; 3.05–3.98 per 100 PYO] and it was significantly higher among adolescents than children [4.86 (4.08, 5.74) vs 2.27 (1.77, 2.86)] per 100 PYO. When stratified by residence, sex, Hgb level, CD4 count and WHO clinical stage, being in the adolescent age was associated with significantly shorter time to LTFU in all strata except CD4>350.

**Fig 2:**
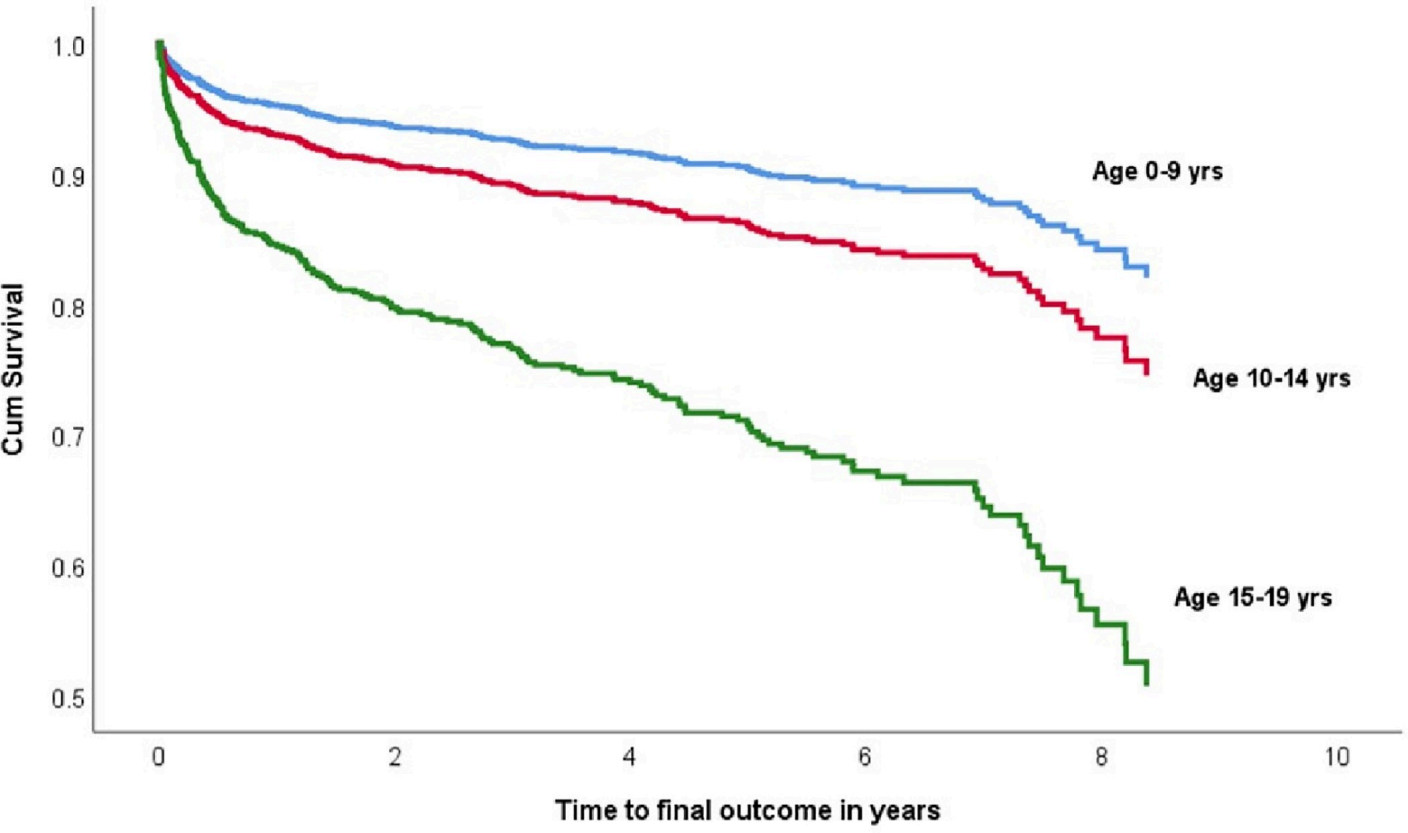
Time-to-loss to follow up by age category after adjusting for potential confounders.

In an unadjusted cox-regression analysis, being in adolescent age group was associated with higher LTFU rates compared with children [crude hazard ratio, cHR (95% CI) = 2.0 (1.5–2.0)]. When further stratified within adolescent age band, older adolescents had the highest hazard of LTFU [(cHR = 3.4 (2.4–4.8)]. Other factors associated with increased LTFU rates in an unadjusted analysis included being from rural area, low CD4 count, and female sex. After adjusting for these potential confounders, being in the older adolescent age group remained a significant predictor of LTFU [adjusted HR, aHR.(95% CI) = 3.1 (2.1–5.0)]. Table 3 summarizes adjusted and unadjusted analyses for potential predictors of LTFU. This is further depicted in Fig 2.

**Table 3:** Predictors of LTFU after ART initiation among adolescents and children (n = 1531).

Variable		cHR (95% CI)	aHR (95% CI)
Age group			
	0–9 yr	Ref	Ref
	10–14 yr	1.5 (1.1, 2.1)	1.5 (0.9,2.3)
	15–19 yr	3.4 (2.4, 4.8)	3.1 (2.1, 5.0)
**Residence**			
	Urban	Ref	Ref
	Rural	2.9 (1.9, 4.3)	3.2 (2.1, 5.0)
**CD4 at ART initiation (count/ml)**			
	> = 350	Ref	Ref
	<350	2.8 (1.8, 4.3)	1.6 (1.01, 2.7)
**Hgb at ART initiation**			
	> = 10g/dl	Ref	Ref
	<10g/dl	1.3 (0.9, 1.9)	1.4 (0.9,2.1)
**Sex**			
	Male	Ref	Ref
	Female	1.5 (1.2, 2.0)	1.5 (1.1,2.1)

cHR = Crude Hazard Ration

aHR = Adjusted Hazard Ratio

**Fig 3:**
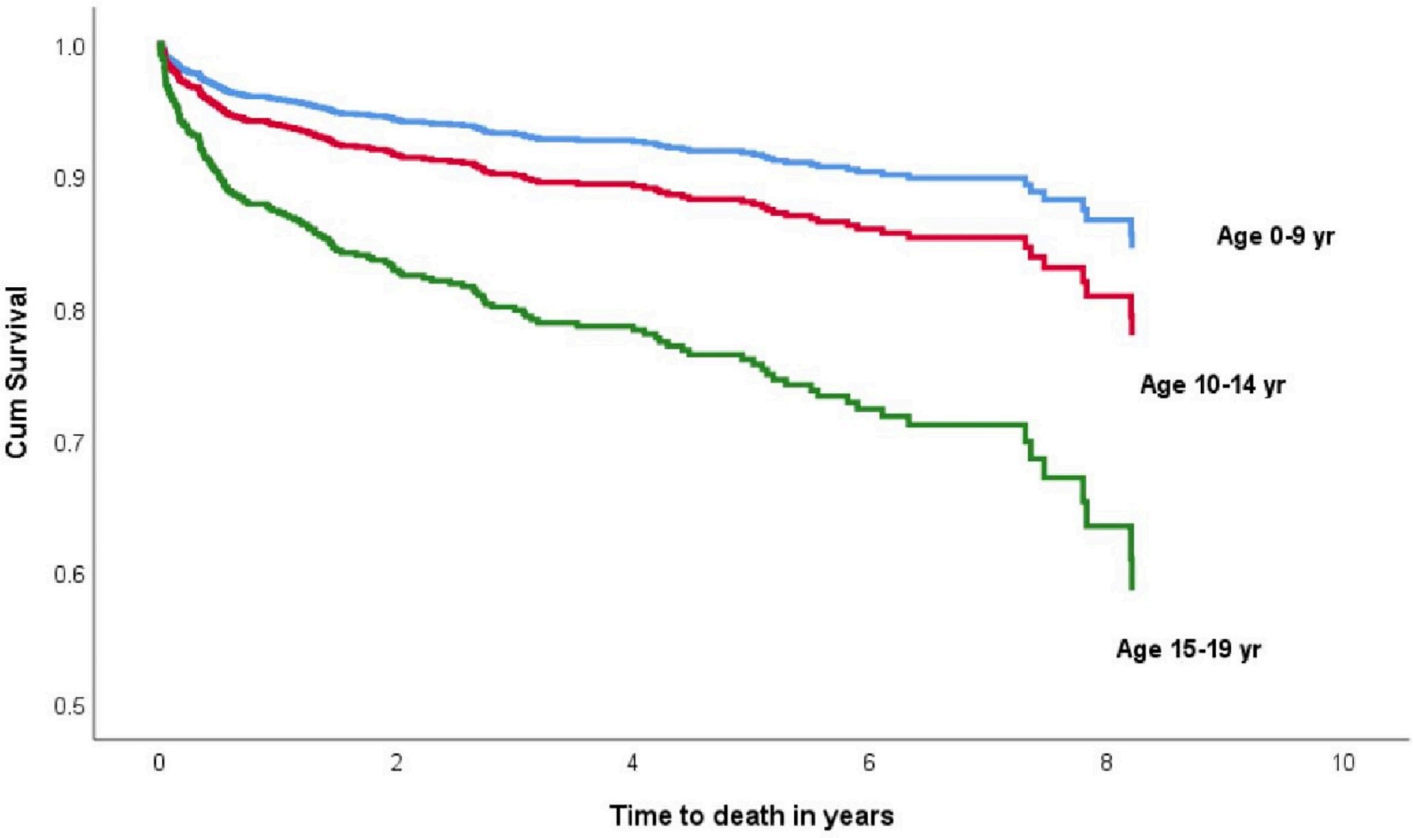
Time-to-death by age category after adjusting for potential confounders.

The overall mortality rate (95% CI) was 1.54 (1.24, 1.89) per 100 PYO (92 deaths per 5984.04 PYO). Mortality was significantly higher in adolescents [2.29 (1.77, 2.92) per 100 PYO] than in children [0.86 (0.57, 1.25)] per 100 PYO. When stratified by residence, sex, Hgb level, CD4 count and WHO clinical stage, being in the adolescent age was associated with significantly shorter time to death in all strata except CD4>350 where the number of events was too few (five) to make comparison. Upon further age stratification, the hazard of progression to death was highest in the younger adolescents in adjusted analyses [aHR = 2.8 (1.4,5.4)] followed by the rate in older adolescents. Table 4 summarizes predictors of mortality which is also depicted in Fig 3.

**Table 4:** Predictors of post-ART mortality according to Cox regression analyses.

Variable		cHR (95% CI)	aHR (95% CI)
**Age group**			
	0–9 yr	Ref	Ref
	10–14 yr	2.4 (1.5,3.9)	2.8 (1.4,5.4)
	15–19 yr	2.4 (1.4,4.3)	2.3 (1.1,4.9)
**Residence**			
	Urban	Ref	Ref
	Rural	2.5 (1.3,4.7)	2.8 (1.3,6.2)
**CD4 at ART initiation (count/ml)**			
	> = 350	Ref	Ref
	<350	5.5 (2.2,13.6)	2.8 (1.1,7.4)
**Hgb at ART initiation**			
	> = 10g/dl	Ref	Ref
	<10g/dl	1.9 (1.1,3.4)	2.1 (1.2,3.8)
**Sex**			
	Male	Ref	Ref
	Female	1.1 (0.7,1.7); p = 0.6	-
WHO stage			
	I-II	Ref	Ref
	III-IV	2.0 (1.2, 3.4)	1.8 (0.9,3.4)
Region			
	Addis Ababa	Ref	Ref
	SNNPR	1.6 (1.1, 2.4)	1.2 (0.6, 2.1)

cHR = Crude Hazard Ration

aHR = Adjusted Hazard Ratio

Out of the 92 deaths reported, 62 (67.4%) occurred during the first year of follow-up. This early mortality rate was more pronounced in the adolescents (46/65, 70.8%) compared with that of children (16/27, 59.3%) and the difference was statistically significant (p<0.001). On the other hand, adolescents and children differed with regard to the proportion lost to follow. Out of 71 children reported to be lost to follow, 40 (56.3%) were lost after the first year of follow-up while in adolescents 61/138 (44.2%) were lost during the first year of follow-up.

## Discussion

Adolescents living with HIV were twice more likely to be LTFU from HIV care compared with children under 10 years of age. Further stratified analyses by age group showed a three-fold increase in LTFU among adolescents aged 15 years and older compared with children. Also, being female, having a rural residence and lower CD4 count at baseline predicted higher LTFU rates. Similarly, being in the adolescent age group was a significant predictor of mortality, with adolescents in the age category 10–14 years being at highest risk of morality irrespective of the place of the region where they received treatment. There was no difference in mortality rate between younger adolescents and children.

Our results provide further evidence supporting the need for age-specific interventions to improve the retention of adolescents and children in care. The higher attrition and mortality rates among girls and rural residents also suggests the need for more in-depth analysis of specific factors leading to higher attrition rates in this sub-set of adolescent population groups. These findings also warrant the need to explore gender-sensitive approaches for adolescents living with HIV in Ethiopia.

Earlier studies on the rates of LTFU and death provided varied results [11, 12, 14] but some recent reports from resource-limited settings provide ample opportunities to compare our results with existing data. In a cohort study in South Africa, for example, there was no difference in rates of mortality and LTFU between adolescents and young adults [18]. A more recent study from Zimbabwe, however, provided more detailed insights about the effect of narrow age bands on LTFU and mortality rates [19]. When stratified by age groups 5–9, 10–14, 15–19 and 20–24 at ART initiation, LTFU rate was highest among the oldest age group but it was comparable between young adolescents and children. and lowest among young adolescents. Although we have used slightly different age band groups in our analysis, the highest LTFU rate among the oldest age group was comparable. Our findings further highlight the importance of narrow age-banding in better understanding age-specific LTFU rates.

The rate of LTFU varied considerably across studies, ranging from as low as 5% at 1 year of follow-up in a Ugandan cohort of adolescents [20] to 32% at four years of follow up in the same country [21]. The duration of follow up for the cumulative LTFU is similar to that of ours but the proportion lost to follow up at a median of 3.8 years was about a half the magnitude reported in the recent Ugandan cohort. In the most recent global collaborative cohort report, the LTFU rate was 13.2% for Sub-Saharan Africa and mortality was reported to be 2.9% for the same sub region [10]. Ethiopia was included in this global cohort data but country-specific information was not reported and the age group was limited to 10–14 years, presuming they would represent perinatally infected adolescents. Further reports on Sub-Saharan cohorts of the same age group showed no significant difference in mortality hazards between low income and upper middle-income countries [15].

Age and sex were examined as important predictors of mortality and LTFU in two recent reports from Sub-Saharan Africa, one from Tanzania and the other as part of the Collaborative Initiative for Paediatric HIV Education and Research (CIPHER) collaborative cohort [15, 22]. Among the Tanzanian cohort, late adolescents are more likely to be lost to follow-up irrespective of their gender but about 80% of girls presented at late adolescence. On the other hand, the CIPHER collaborative analysis showed that there was no difference in mortality among males and females. Our findings further confirm the lack of mortality differences by gender. However, the higher LTFU among girls in our study could be related to broader socio-cultural factors that disfavor the female child in the African society but recent systematic reviews suggest inconsistencies across studies [23]. The route of infection is an important factor that needs to be considered as potential contributor to the high mortality in the 10–14 age group and the high LTFU in the 15–19 age group. It might also explain the higher LTFU in the females, if a greater proportion of adolescent females were recently rather than perinatally infected. This area needs further exploration and more in-depth analysis.

Predictors of higher mortality appear to be consistent across pediatric age groups. As recently reported by our team, anemia and rural residence were significant predictors of mortality both in children and young adults [24]. The predictive value of anemia as a marker of disease severity and eventual mortality is a well-established fact even among adult patients [25]. We reconfirm the utility of this relatively simple marker for prioritizing patient care. Being from a rural area could also be an indirect marker of poorer quality of care in remote parts of the country. It could also be due to unascertained mortality in rural areas which was reported in 40.6% of LTFU adult patients traced in southern Ethiopia [17].

Poor retention in care is recognized as a global challenge for adolescents and young adults living with HIV, suggesting the need for a comprehensive package of targeted interventions to address them [26]. In a recent rapid review, a range of interventions were suggested to be effective including cognitive behavioral therapy, education, the use of adherence supporters, directly observed therapy, and active adherence reminder devices [27]. More recent and comprehensive cohort data also identified high pre-treatment attrition rates especially among infants and older adolescents, calling for tailored interventions to address this challenge [28]. Another report from the same cohort identified worse mortality and LTFU rates among older adolescents [29]. The three-fold increase in LTFU concurs with this latest global cohort report but the lack of difference in mortality between younger and older adolescents needs further investigation. The higher LTFU among older adolescents could be associated with change of school address for healthier adolescents. Other possible explanations include adolescents dropping out of care due to stigma and discrimination which is a common challenge in the Ethiopian setting [30]. This area requires more in-depth studies for the different age groups.

Our study findings also highlight additional dimensions to be considered while planning for larger scale implementation studies. Female sex, rural residence, more advanced immunosuppression at initiation and specific age band should be included as potential predictors of future studies. Interventions to reduce LTFU may need to specifically address the challenges faced by vulnerable adolescents with these risk factors.

This study has important limitations which should be taken into consideration while interpreting the findings. Firstly, since viral load measurement was not done routinely and immunologic parameters were measured inconsistently, we were not able to determine virologic and immunologic parameters both as predictors and outcomes of the cohort. Secondly, the retrospective nature of data collection did not allow us to include important potential predictors of outcomes such as routes of HIV transmission. Also, we were not able to trace and determine the final outcomes of those reported as LTFU in the medical records due to resource limitations. The other limitation is that we did not consider current age in the analysis which may have provided further insights about the effect of age on patient attrition. However, the large sample size and rigorous statistical methods used to compare the results are the strengths of the study. Moreover, this is one of a few studies comparing treatment outcomes across three age bands encompassing adolescents and children.

The findings in this study further advance our understanding about the higher risk of attrition from ART programs among adolescents living with HIV and receiving ART. The narrow age bands used in this study can be adapted to programmatic settings to help tailor interventions to the unique needs of each age group. Further, we now have clear evidence that girls are at higher risk of being LTFU compared with boys. While this study has provided important insights into the magnitude and predictors of mortality and LTFU among adolescents, further studies are needed to better understand factors that put adolescents at higher risk of being lost to follow up. We also suggest that a package of interventions be developed, tested and implemented based on findings from this and further studies.

## Supporting information

S1 File S1_Minimal dataset after reviewer comment.sav. (SAV)
